# A Literature Review of Stainless Steel Crown for Permanent Molars: Indications, Survival, Periodontal and Radiographic Findings

**DOI:** 10.30476/dentjods.2024.101475.2301

**Published:** 2025-03-01

**Authors:** Pooya Vatankhah, Fatemeh Hashemi, Alireza Sarraf Shirazi

**Affiliations:** 1 Student Research Committee, School of Dentistry, Mashhad University of Medical Sciences, Mashhad, Iran; 2 Dept. of Pediatric Dentistry, School of Dentistry, Mashhad University of Medical Sciences, Mashhad, Iran

**Keywords:** Crowns, Dentition, Permanent, Molar, Pediatric Dentistry

## Abstract

Stainless steel crowns (SSCs) are the preferred choice for restoring primary molars with extensive caries. However, they may be indicated for permanent molars in certain cases as well. While limited research assessed different aspects of this treatment separately, this review aims to consolidate the existing literature and provide a multi-dimensional analysis of preformed metal crown restoration for permanent molars. A comprehensive search of electronic databases including PubMed, Scopus, Web of Science, Embase, and Cochrane was conducted, and relevant studies were categorized based on specific aspects of this treatment including indications, survival rates, periodontal and radiographic findings, utilization frequency, and alternative options. The review highlights the wide range of indications for SSCs on permanent molars of children, adolescents, and even adults, along with their remarkable success rates. However, noticeable underutilization, despite their proven efficacy, was observed. Periodontal defects and marginal discrepancies were found to be the most common causes of failures.

## Introduction

Since their introduction by Engel [ 1 ] in the 1950s, stainless steel crowns (SSCs) have remained as a treatment option for restoring primary molars with extensive multi-surface caries [ 2 ]. These prefabricated metal crowns are fitted to a single tooth and cemented with proper luting agent. Over the years, numerous studies have shown that SSCs may provide a longer lifespan compared to other restorative options for primary teeth. These crowns also have favorable mechanical properties, can be placed in a single appointment, reduce the risk of recurrent caries, help maintain occlusal function and periodontal health, and retain the arch length [ 3 - 6 ]. Consequently, the American Academy of Pediatric Dentistry (AAPD) mentions that SSC is preferred for primary molars with extensive carious lesions and severe developmental defects [ 7 ].

Dental caries in permanent molars is a common occurrence across different age groups. While there are several treatment options available to restore damaged tooth structure, the severity of caries sometimes necessitates the use of full-coverage restorations. For permanent molars with significant tooth structure loss, custommade crowns should be considered as the definitive restoration to reduce the risk of tooth fracture [ 8 ]. However, definitive permanent crowns may not always be the optimal choice for such cases. Depending on various circumstances such as incomplete jaw growth, uncooperative patients, developmental defects and more, dentists may need to consider alternative treatment options, including the use of preformed crowns such as SSC. The indications will be further explored and discussed later in the article.

Limited research has focused on SSC restoration for permanent molars, and previous studies have assessed various aspects of this treatment separately. This study aims to consolidate the existing literature, providing a multi-dimensional review of indications, survival rates, periodontal and radiographic findings, utilization frequency, and alternative options to this treatment. This review aims to offer a clearer perspective to dental practitioners who may encounter such cases.

## Search Strategy

A comprehensive search of electronic databases, including PubMed, Scopus, Web of Science, Embase, and Cochrane, was conducted up to October 28th, 2023. The aim was to identify English-language studies related to SSCs and permanent dentition using a specific search strategy. Refer to Table 1 for more details.After the consolidation of articles retrieved from all the mentioned databases, duplicate studies were removed using EndNote 20 software. To identify eligible studies, the titles and then the abstracts of the identified articles were screened by two independent reviewers. In the next step, the full texts of the relevant studies were screened for eligibility. The authors were reached out to obtain the full texts of the pertinent abstracts whenever feasible. Any disagreements were resolved through discussion and consensus.

**Table 1:** Conducted search strategy to identify relevant articles

Search Term	Database	Results
“((Permanent Dentition) OR (Secondary Dentition) OR (Adult Dentition) OR (permanent AND (oral OR mouth OR teeth OR tooth OR dent* OR maxilla* OR mandib* OR molar* OR incis* OR palatal* OR lingual)) AND ((Stainless steel crown) OR SSC)”	PubMed	338
	Scopus	341
	Web of Science	103
	Embase	241
	Cochrane	68

Total: 1091, After duplicate studies removal: 523

## Results

From the 35 identified articles after the screening process, case-reports, non-English articles, and those without available full-texts were subsequently excluded from the analysis, resulting in a total of 26 studies deemed eligible for this review. This selection included both interventional and observational studies that evaluated various aspects of SSC restoration for permanent molars. Additionally, questionnaires regarding the usage of it and those indicating alternative approaches were also included. Given the narrative nature of this review and variety of study designs, no formal quality assessment form or statistical analysis was employed. However, the reviewers critically appraised the included studies for methodological rigor, study design and potential sources of bias.

## Literature Review

The gathered information about SSC restoration for permanent molars was categorized into the following groups: Indications, Success Rate, Periodontal Findings, Radiographic Findings, Utilization Frequency, and Alternative Options. It is important to note that the articles within each category are not entirely distinct, as some studies may cover multiple aspects of SSC restorations. This overlap means that certain articles appear in more than one category, reflecting the multifaceted nature of the research on this topic.

## Discussion

### Indications

In a 2023 study conducted by Stoica *et al.* [ 9 ], it was reported that 60.9% of children in the mixed dentition stage had at least one first permanent molar affected by caries. Direct restorative materials like amalgam and resin restorations have demonstrated lower survival rates in cases with extensive caries [ 5 , 10 ]. The use of definitive crown restorations on partially erupted teeth with short clinical crowns can lead to inadequate retention. Additionally, due to gradual positional changes, alterations in the margin of restoration and occlusion can occur. An interim SSC may offer a more suitable choice for these cases [ 6 , 11 - 12 ]. Delaying the placement of permanent custommade crowns until the tooth position and occlusion have been fully established, typically around the age of 18, is recommended [ 12 ].

Molar-incisor hypomineralization (MIH) is the most common developmental enamel defect that affects children and adolescents. Clinically, MIH is characterized by distinct opacities of varying sizes, which can appear discolored in shades ranging from white to yellow-brownish. It typically involves at least one permanent first molar, with or without affecting the incisors as well [ 13 ]. Managing these cases can be quite complex due to the heightened risk of caries development, tooth hypersensitivity, fragile enamel, and esthetic concerns, particularly if the incisor areas are involved. The choice of treatment can range from methods such as remineralization, sealing, and resin-based or amalgam fillings, to more extensive approaches like SSCs and in severe cases, tooth extraction [ 13 - 16 ]. Until now, there has been no agreement on the preferred treatment approach for such cases. However, SSCs and resin restorations have been consistently popular choices over the years. Although composite restorations are recommended for well-defined defects involving up to three surfaces, SSCs have demonstrated higher success rates in molars affected by MIH [ 17 - 19 ]. Nonetheless, when deciding on treatment, clinicians must consider a range of patient factors (age, cooperation, caries risk, expectations), defect characteristics (extent, severity, location, symptoms), and material considerations (cost, longevity, aesthetics) [ 18 ].

Amelogenesis imperfecta (AI) and dentinogenesis imperfecta (DI) refer to inherited disorders in enamel and dentin formation affecting both primary and permanent dentition [ 20 - 21 ]. Due to the potential association of these conditions with other intraoral disorders, AAPD emphasizes the necessity of interdisciplinary collaboration for better management of these cases in the permanent dentition [ 22 ]. While SSCs are frequently utilized as the preferred restoration for primary molars affected by AI or DI, they can serve as an appropriate interim solution for such permanent molars as well.It is worth noting that this treatment is not exclusive to a particular age group. Uncooperative adult patients with special needs, such as intellectual disability, autism, seizures, cerebral palsy, physical impairment, and genetic abnormalities may require the administration of this treatment under general anesthesia. Additionally, it presents an affordable restorative option for individuals with limited financial resources, those in remote areas without regular dental care access, as well as the elderly. Other than extensive decays and developmental tooth defects, it can also offer a provisional coronal restoration for traumatized teeth with an uncertain prognosis [ 23 - 25 ]. 

### Success Rate

In recent years, there has been an increasing focus on the success rate of this treatment. A summary of these can be observed in Table 2. Interpreting and generalizing the results based on different patients and age groups, different therapists, and varying criteria for success or failure should be done with caution. However, notable similarities also exist.

**Table 2:** Data retrieved from the relevant articles regarding the survival rate

Author, Year	Country	Number of Participants	Age	Objective	Results
Geduk *et al.* 2023 [ 42 ]	Turkey	17 patients	Average age of 8 years (6-13 years)	To assess the 18 months survival rate of SSCs in comparison to preformed zirconia crowns	100% survival rate for 20 SSC and 95% for 20 preformed zirconia crown restorations
Chaipattanawan *et al.* 2022 [ 12 ]	Thailand	99 patients	Average age of 9.6 years (6-16 years)	To assess the average 33.5 months survival rate of SSCs (with a range of 6-104 months follow-up)	64.3% survival rate for 140 SSC restorations
de Farias *et al.* 2022 [ 18 ]	Colombia	115 patients	Mean age of 8.4 years (7-10 years)	To assess the 24 months survival rate of SSCs in comparison to composite resin restorations in molars affected by MIH	94.4% survival rate for 54 SSC restorations, 49.2% for 61 composite resin restorations
Felemban *et al.* 2021 [ 26 ]	Saudi Arabia	23 patients	Mean age of 11.75 years (10-15 years)	To assess the survival rate of SSCs placed from 2015 to 2018	86.1% survival rate for 36 SSC restorations
Sigal *et al.* 2020 [ 23 ]	Canada	271 patients with special needs	Median age of 44 years (15.5-81.9 years)	To assess the 10-year survival rate of SSCs in comparison to amalgam and composite resin restorations	79.2% survival rate for 650 new SSC, 63.5% for 1011 new amalgam, 37% for 201 preexisting composite resin restorations
Discepolo *et al.* 2017 [ 11 ]	United States	155 patients	Between 6-19 years	To assess the average 45 months survival rate of SSCs (with a range of 6-99 months follow-up)	88.3% survival rate for 155 SSC restorations
Kotsanos *et al.* 2005 [ 17 ]	Greece	36 patients	Mean age of 7.7 years	To evaluate the restorative management for patients diagnosed with MIH	100% survival rate for 24 SSC restorations after 52 months, 74.6% for 59 composite resin, 77.1% for 35 sealant, and 38.9% for 18 amalgam restorations

In Sigal *et al.* [ 23 ] study, 91 (11.9%) out of 766 SSCs have failed. Of which, 35 (38.5%) needed recommendation or replacement and 56 (61.5%) required extraction. The most common causes leading to the extraction of SSC-restored teeth were periodontal defects, followed by pulpal pathology and caries respectively. These findings are supported by Discepolo *et al.* [ 11 ] and Felemban *et al.* [ 26 ] studies, as the most common cause of failure was periodontal defects. Chaipattanawan *et al.* [ 12 ] reported that the primary reason for failures, accounting for 34%, was the marginal discrepancy caused by continuous tooth eruption, resulting in supragingival shifting of the margin and the formation of “small ledges”. They also found that periodontal defects contributed to 18% of the failures. By evaluating associated risk factors, loss of proximal walls and unsatisfactory immediate post-operative condition were identified as probable causes for these failures.

Moehn *et al.* [ 27 ] found that SSCs might be more prone to occlusal wear and subsequent perforation compared to other prefabricated crowns. However, occlusal perforation was not identified as a major cause of failures and could be easily resolved through crown replacement or amalgam repair [ 23 ]. In terms of pulpal failures, Linas *et al.* [ 28 ] evaluated the outcomes of complete pulpotomy treatment using zinc oxide-eugenol pulp capping material on permanent molars that were immediately restored with SSCs. After 24 months, 234 (89%) out of 263 pulpotomies were deemed effective, while 7.6% were uncertain and 3.4% were ineffective.

The impaction of adjacent teeth (26%) was reported as the second main cause of failures by Chapattanawan *et al.* [ 12 ], possibly due to overextended or open margins. It is worth noting that by implementing a few key measurements to ensure the proper marginal fit of SSCs on permanent molars, the majority of the primary causes of failures mentioned earlier, such as marginal discrepancies, periodontal defects, impaction of adjacent teeth, and recurrent caries, can be effectively addressed and prevented to some extent. These measurements may involve selecting the appropriate crown size, trimming it to the proper length, crimping the edges, thoroughly finishing and polishing the margins, and carefully evaluating the fit of the crown before cementation, both clinically and radiographically [ 29 - 30 ]. Furthermore, it has been reported that substituting conventional glass ionomer cement with resin-modified glass ionomer cement may contribute to the reduction of the marginal gap [ 31 ].

### Periodontal Findings

As mentioned earlier, several studies have indicated the significance of periodontal conditions and marginal discrepancies in the failure of SSCs in permanent molars.

The objective of this section is to delve into the clinical periodontal findings of this treatment in greater detail.

In a study conducted by Heidari *et al.* [ 32 ] in 2019, periodontal indices were assessed in permanent molars restored with SSCs after a 6-month treatment period. The findings revealed a significant reduction in pocket depth on the mesiobuccal and mesiolingual surfaces, which could be attributed to children's ability to properly clean these surfaces, as they are more accessible. This highlights the importance of maintaining proper oral hygiene for the periodontal health of SSCs, considering their preformed nature and potential for low marginal adaptation. The study also noted a significant decrease in bleeding on probing and an improvement in gingival color. Additionally, the control groups demonstrated a reduction in these indices, further emphasizing the favorable effects of oral hygiene instructions, which were provided to all participants.

Gandhi *et al.* [ 33 ] measured inflammatory cytokines in the gingival crevicular fluid (GCF) of primary and permanent molars that were restored with SSCs after one month of treatment. The concentration of macrophage inflammatory proteins 1α and 1β (MIP-1α and MIP-1β), which serve as biomarkers for determining underlying inflammation, was found to be increased in the GCF. These findings are supported by a study conducted by Koleventi *et al.* [ 34 ] which assessed the gingival index, probing depth, and the count of two periodontal pathogens (*Tannerella forsythia* and *Porphyromonas gingivalis*) after 6 months of restoring permanent teeth with SSCs. The study noted a statistically significant increase in both the indices and subgingival microbial counts; even though the SSCs exhibited well-fitting margins and no overhangs were observed in the restorations.

According to reports, a marginal fit of 120μm is considered clinically acceptable [ 35 ]. However, achieving such a marginal gap is possible only with custom crowns, not with preformed crowns like SSCs. While no other study has specifically assessed the fit of SSCs for permanent molars, it is worth noting that in the case of primary molars, as expected, the adaptation of SSCs deviated from the ideal fit mentioned for custom crowns [ 31 ]. Although SSCs, even when properly fitted on permanent molars, showed an increase in periodontal indices and inflammatory biomarkers, Heidari *et al.* [ 32 ] demonstrated that these changes could be addressed to some extent by maintaining proper oral hygiene.

### Radiographic Findings

Indeed, to properly assess the treatment outcomes, it is essential to conduct both clinical and radiographic examinations. This section aims to highlight the most important radiographic findings and their prevalence in relation to SSC-restored permanent molars.

Munoz-Sanchez *et al.* [ 36 ] conducted a retrospective study to collect and assess radiographic images of 360 SSC restorations in permanent teeth. The study involved 198 adult patients who received treatment under general anesthesia for an average follow-up period of 8.9±14.3 months. Four criteria were used to analyze the radiographic images, including marginal adaptation, proximal contact with adjacent teeth, presence of glass ionomer cement overflow, and interproximal alveolar bone loss. The bone loss was assessed by comparing the bone situation in the radiographic image taken during the anesthesia session with the latest follow-up radiograph. The remaining three criteria were evaluated using the latest follow-up radiograph.

Most of the major defects observed in radiographs were primarily associated with marginal adaptation, affecting 37.5% of the SSC restorations. Interproximal contact defects (17.8%) and cement overflow defects (4.2%) were less frequently observed. An association was found between alveolar bone loss and interproximal contact defects on both mesial and distal surfaces. However, there was no correlation between bone loss and either cement overflow defects or marginal adaptation defects. Overall, a satisfactory marginal adaptation was reported to be less achievable, consistent with the study conducted by Chaipattanawan *et al.* [ 12 ], which identified it as the primary cause of failures.When analyzing 216 teeth for alveolar bone loss, major periodontal defects were observed in 14 (8.3%) of them. The study by Sigal *et al.* [ 23 ] reported a mean bone loss of 1.36mm and 1.40mm on the mesial and distal surfaces of 254 permanent molars restored with SSCs in adult patients. Munoz-Sanchez *et al.* [ 36 ] reported a low risk of medium-term periodontal morbidity in SSCs placed on permanent molars. However, they noted that the results should be interpreted cautiously as it was solely based on radiographic criteria. Furthermore, the mean follow-up time may have been shorter than the time needed to recognize a marked periodontal defect accurately. However, in other studies involving younger patients, Discepolo *et al.* [ 11 ] found that 38% of failed SSCs on permanent molars, after an average of 45 months, were associated with vertical or severe horizontal (over 5mm) bone resorption. Additionally, Chaipattanawan *et al.* [ 12 ] reported that 14% of failed SSCs on permanent molars, after an average of 33.5 months, exhibited marked bone loss in the coronal third of the root.

It is important to note that in patients with mixed dentition, there is another crucial aspect that should be carefully evaluated the impaction of adjacent teeth. Chaipattanawan *et al.* [ 12 ] found that 26% of failures and Discepolo *et al.* [ 11 ] found that 11% of failures were attributed to this issue. This review previously discussed possible reasons and ways to prevent this problem.

In addition to the mentioned radiographic criteria, the presence of periapical pathology is the first thing to evaluate in radiographic images of such cases. While achieving an ideal marginal seal with preformed crowns is more challenging compared to custom crowns, none of the studies reported endodontic failures as a significant concern in terms of treatment outcomes. However, the presence of a periapical lesion in the radiograph of such teeth indicates a definite failure and necessitates a more comprehensive treatment plan to preserve the tooth.

### Utilization Frequency

This section will investigate the case scenarios and the treatment choices made by dental practitioners through conducted studies. By doing so, the usage frequency and perspectives of healthcare providers towards this treatment, along with the associated reasons will be explored.

In two separate retrospective studies [ 25 , 37 ], the prevalence of SSC restorations performed for maxillary and mandibular first permanent molars among Indian children and adolescents was assessed. Only 6% of the patients underwent SSC treatment for their mandibular first permanent molars [ 25 ]. The usage was even lower at 1% for maxillary first permanent molars [ 37 ].

In a study by Kopperud *et al.* [ 38 ], a questionnaire was administered to Norwegian dentists to determine their preferred restoration for a partially erupted first permanent molar with severe damage due to MIH. The author suggested that SSC would be the better choice for retaining the tooth. However, interestingly, only 11% of the dentists selected SSC. The majority of the dentists preferred using glass-ionomer cement and composite restoration, respectively [ 38 ].

Similar results were observed when Wuollet *et al.* [ 39 ] and Uhlen *et al.* [ 40 ] surveyed dentists in Finland and Norway about a hypomineralized and grossly broken down first permanent molar in a 9-year-old patient. SSCs accounted for only 10% and 5% of the selected restorations.

Considering all these findings collectively, it is clear that there is a noticeable underutilization of this treatment for permanent molars, despite its superior lifespan compared to direct restoration materials. Previously, there have been reports of underuse of SSCs for restoring primary molars [ 4 , 41 ]. However, it appears that the frequency of utilization of this treatment for permanent molars may be even lower than when it is deemed necessary. This could be attributed to inadequate training, insufficient clinical expertise, and limited knowledge regarding the extensive range of indications for SSCs in permanent molars.

### Alternative Options

A brief overview of the success rates of different direct restorative materials compared to SSCs in permanent molars has been provided in Table 2. However, it is crucial to recognize that each material may have specific indications. The purpose of this section is to explore other innovative approaches to manage such cases.

Zirconia crowns are commonly used as preformed crowns for restoring primary molars with extensive damage. Numerous studies have compared various aspects of preformed zirconia and metal crowns as restorations in primary molars. In recent years, with an increasing focus on aesthetics, researchers have also evaluated the utilization of zirconia crowns for permanent molars in special cases as well.

In 2023, Geduk *et al.* [ 42 ] conducted a study to assess the performance of SSCs and preformed zirconia crowns in 40 permanent molars of children with mixed dentition. The evaluation took place over an 18-month follow-up period. The survival rate of SSCs was found to be 100%, while zirconia crowns had a survival rate of 95%. Teeth restored with zirconia crowns exhibited significantly lower gingival index and plaque index scores compared to those restored with SSCs. It is worth noting that no clinically unacceptable marginal discrepancies were observed in any of the restorations. Similar findings were observed in primary molars as well. Donly *et al.* [ 43 ] reported that gingival health adjacent to primary molars restored with zirconia crowns was significantly better compared to those restored with SSCs. The improved gingival health observed in primary and permanent molars restored with zirconia crowns can be attributed to the smooth and glazed surface of these restorations, which reduces the roughness and the retention of dental plaque [ 42 ]. Additionally, when used with glass ionomer cement, zirconia crowns resulted in the lowest dentinal stress in permanent molars compared to SSCs and other bonding agents [ 44 ]. This fact should be taken into consideration by dental practitioners when dealing with a permanent molar that is affected by developmental defects and has a fragile dentin, as there is a possibility of future fracture.Despite the favorable outcomes and superior esthetics of preformed zirconia crowns for permanent molars, it is important to note that they are more expensive and require nearly twice the amount of time to perform compared to SSCs [ 45 ]. Therefore, SSCs may be a more suitable option for restoring permanent molars in uncooperative patients or those with limited financial resources. Additionally, the placement of zirconia crowns requires a passive fit and may result in the loss of more tooth structure during preparation [ 46 ]. Hence, careful consideration should be given to the preparation of such permanent molars if a definitive crown restoration is indicated in the future.With the recent advancements in digital and adhesive dentistry, researchers are exploring new approaches to managing these cases. While SSCs are not typically viewed as a definitive treatment for young molars affected by MIH, indirect composite and CAD-CAM restorations offer a more definitive approach with improved aesthetics and less invasive tooth preparation [ 47 - 48 ]. In a one-year follow-up, indirect composite restorations showed nearly identical success rates to direct composite restorations in molars affected by MIH. Additionally, they reported higher child satisfaction levels due to shorter treatment sessions [ 48 ]. In a 2023 study conducted by Eldehna *et al.* [ 49 ] CAD-CAM zirconia and IPS e.max overlays also demonstrated a favorable success rates for hypomineralized permanent molars after a year. However, further exploration of the long-term success of these alternative options and a direct comparison to SSCs is recommended for future research.

## Conclusion

SSCs have a wide range of indications for permanent molars in children, adolescents, and even adult patients, demonstrating remarkable success rates when compared to alternative options. However, there is a notable underutilization of this treatment, even when it is deemed necessary.Despite variations in study designs, periodontal defects and marginal discrepancies were identified as the most common causes of failures. These issues, though partly preventable, are especially crucial for permanent molars as they would serve for many years and may require a definitive crown later on.
